# New chromosome counts and genome size estimates for 28 species of *Taraxacum* sect. *Taraxacum*

**DOI:** 10.3897/CompCytogen.v12i3.27307

**Published:** 2018-09-18

**Authors:** Petra Macháčková, Ľuboš Majeský, Michal Hroneš, Eva Hřibová, Bohumil Trávníček, Radim J. Vašut

**Affiliations:** 1 Department of Botany, Faculty of Science, Palacký University in Olomouc, Šlechtitelů 27, 783 71, Olomouc, Czech Republic Palacký University in Olomouc Olomouc Czech Republic; 2 Institute of Experimental Botany, Centre of the Region Haná for Biotechnological and Agricultural Research, Šlechtitelů 31, 779 00 Olomouc, Czech Republic Centre of the Region Haná for Biotechnological and Agricultural Research Olomouc Czech Republic

**Keywords:** Asteraceae, chromosome number, flow cytometry, karyology, *
Taraxacum officinale
*

## Abstract

The species-rich and widespread genus *Taraxacum* F. H. Wiggers, 1780 (Asteraceae subfamily Cichorioideae) is one of the most taxonomically complex plant genera in the world, mainly due to its combination of different sexual and asexual reproduction strategies. Polyploidy is usually confined to apomictic microspecies, varying from 3x to 6x (rarely 10x). In this study, we focused on Taraxacum sect. *Taraxacum* (= T.sect.Ruderalia; *T.officinale* group), i.e., the largest group within the genus. We counted chromosome numbers and measured the DNA content for species sampled in Central Europe, mainly in Czechia. The chromosome number of the 28 species (*T.aberrans* Hagendijk, Soest & Zevenbergen, 1974, *T.atroviride* Štěpánek & Trávníček, 2008, *T.atrox* Kirschner & Štěpánek, 1997, *T.baeckiiforme* Sahlin, 1971, *T.chrysophaenum* Railonsala, 1957, *T.coartatum* G.E. Haglund, 1942, *T.corynodes* G.E. Haglund, 1943, *T.crassum* H. Øllgaard & Trávníček, 2003, *T.deltoidifrons* H. Øllgaard, 2003, *T.diastematicum* Marklund, 1940, *T.gesticulans* H. Øllgaard, 1978, *T.glossodon* Sonck & H. Øllgaard, 1999, *T.guttigestans* H. Øllgaard in Kirschner & Štěpánek, 1992, *T.huelphersianum* G.E. Haglund, 1935, *T.ingens* Palmgren, 1910, *T.jugiferum* H. Øllgaard, 2003, *T.laticordatum* Marklund, 1938, *T.lojoense* H. Lindberg, 1944 (= *T.debrayi* Hagendijk, Soest & Zevenbergen, 1972, *T.lippertianum* Sahlin, 1979), *T.lucidifrons* Trávníček, ineditus, *T.obtusifrons* Marklund, 1938, *T.ochrochlorum* G.E. Haglund, 1942, *T.ohlsenii* G.E. Haglund, 1936, *T.perdubium* Trávníček, ineditus, *T.praestabile* Railonsala, 1962, *T.sepulcrilobum* Trávníček, ineditus, *T.sertatum* Kirschner, H. Øllgaard & Štěpánek, 1997, *T.subhuelphersianum* M.P. Christiansen, 1971, *T.valens* Marklund, 1938) is 2n = 3x = 24. The DNA content ranged from 2C = 2.60 pg (*T.atrox*) to 2C = 2.86 pg (*T.perdubium*), with an average value of 2C = 2.72 pg. Chromosome numbers are reported for the first time for 26 species (all but *T.diastematicum* and *T.obtusifrons*), and genome size estimates for 26 species are now published for the first time.

## Introduction

*Taraxacum* F. H. Wiggers, 1780 (Asteraceae subfamily Cichorioideae) is a species-rich genus of common and widespread perennial grassland herbs growing from the subtropics to subarctic (arctic/alpine) regions across the world. Rough estimates suggest the genus contains approximately 2,800 species in approximately 60 sections (Kirschner et al. 2015), with the higher diversity in the mountains of Eurasia (Ge et al. 2011); a total of 1,900 species in 35 sections are listed for Europe (Kirschner et al. 2007). The complexity of *Taraxacum* taxonomy is caused by its combination of different reproduction strategies, including sexual reproduction (mainly outcrossing, less frequently selfing) and apomixis (meiotic diplospory; Richards 1973, Asker and Jerling 1992, Kirschner and Štěpánek 1994, Kirschner et al. 1994, Majeský et al. 2017). The vast majority of *Taraxacum* taxa are apomictic polyploid microspecies, only a few species are sexual diploids. The phenomenon of apomixis itself (i.e. clonal reproduction by seeds) attracts the attention of plant systematists as well as plant breeders for its possible application in crop breeding.

The basic chromosome number in *Taraxacum* is x = 8, and it is constant across all the sections. The diploid number (2n = 2x = 16) is confined to only sexually reproducing species, and sexual species are nearly all diploids, with only a few exceptions of sexual tetraploids known in section Piesis (Kirschner and Štěpánek 1994, 1998a, Trávníček et al. 2013). In contrast, apomictic species are never diploids but always polyploids (Majeský et al. 2017), having one of the genes involved in regulation of apomixis (*DIPLOSPOROUS*) located on the NOR chromosome (Vašut et al. 2014). Most of the known chromosome numbers for apomictic *Taraxacum* species are at a triploid level (2n = 3x = 24), especially those of the widespread European sections Taraxacum sect. *Taraxacum* (Mártonfiová 2006, Kula et al. 2013), T.sect.Erythrosperma (Małecka 1967, 1969, Vašut 2003, Schmid et al. 2004, Vašut et al. 2005, Uhlemann 2007, 2010, Vašut and Majeský 2015, Wolanin and Musiał 2017), T.sect.Palustria (Małecka 1972, 1973, 1978, Kirschner and Štěpánek 1998b, Marciniuk et al. 2010) and T.sect.Hamata (Mogie and Richards 1983, Øllgaard 1983). However, tetraploids (2n = 4x = 32) also occur quite frequently in some sections, such as the European dandelions in sections T.sect.Palustria (e.g., *T.vindobonense* Soest, 1965, *T.brandenburgicum* Hudziok, 1969 and *T.portentosum* Kirschner & Štěpánek, 1998), T.sect.Erythrosperma (e.g., *T.tortilobum* Florström, 1914, *T.fulvum* Raunkiaer, 1906 and *T.bifurcatum* Hagendijk et al., ineditus), T.sect.Naevosa (e.g., *T.euryphyllum* (Dahlstedt, 1911) M. P. Christiansen, 1940 and *T.naevosum* Dahlstedt, 1900), T.sect.Scariosa and T.sect.Celtica (*T.unguilobum* Dahlstedt, 1912 and *T.fulvicarpum* Dahlstedt, 1927). Higher ploidy levels are uncommon in *Taraxacum*, while natural pentaploids (2n = 5x = 40; e.g., in the European species *T.skalinskanum* Małecka & Soest, 1972 and *T.zajacii* J. Marciniuk et P. Marciniuk, 2012 and 6 other species of section Palustria, *T.faeroense* Dahlstedt in H. H. Johnston, 1926 of T.sect.Spectabilia, *T.caledonicum* A. J. Richards, 1972 of section Celtica and *T.albidum* Dahlstedt, 1907 of section Mongolica from Japan), hexaploids (2n = 6x = 48 for *T.ranunculus* Kirschner & Štěpánek, 1998 of section Palustria and *T.nordstedtii* Dahlstedt, 1911 of section Celtica), and aberrant heptaploid (2n = 7x = 56) or decaploid (2n = 10x = 80) mutants of natural species have been documented (Richards 1969, Małecka 1973, Mogie and Richards 1983, Kirschner and Štěpánek 1984, 1998b, Sato et al. 2011, Marciniuk et al. 2012). The geographic distribution of diploids and polyploids in Europe is more or less separated, with polyploids mainly distributed in the colder regions of mountains in the north and with diploid sexuals distributed in warmer regions of the south, which results in the phenomenon of geographic parthenogenesis (den Nijs et al. 1990, den Nijs and van der Hulst 1988, Uhlemann 2001, Verduijn et al. 2004a).

Genome size estimation (plant genome C-value) (Greilhuber et al. 2005) is a rapid cytogenetic method that helps provide a better understanding of the evolutionary relationships among studied taxa. The method itself has methodological limitations (multiple factors can affect the measurements; the method does not provide any information on repetitive sequences involved; etc.); however, patterns of genome size estimates in species groups provide additional information on possible pathways of evolution (Soltis et al. 2003, Leitch et al. 2005, Šmarda et al. 2012). Although flow cytometry was widely used in *Taraxacum* research for rapidly identifying the ploidy level in mixed apomictic-sexual populations (e.g., Meirmans et al. 1999, Verduijn et al. 2004a, 2004b, Mártonfiová 2006, 2015, Mártonfiová et al. 2007, 2010) or in taxonomic revisions (e.g., Vašut 2003), genome size estimates are very limited. Genome size (C-value) in *Taraxacum* varies (in known species) between 2C = 1.74 pg in diploid *T.linearisquameum* Soest, 1966 and 2C = 6.91 pg in tetraploid *T.albidum* (Záveský et al. 2005, Siljak-Yakovlev et al. 2010); European triploid apomicts have a value of 2C ≈ 2.4–2.76 pg (Bennett et al. 1982, Záveský et al. 2005, Bainard et al. 2011, Iaffaldano et al. 2017). Considerable variation (~1.2-fold difference) in DNA content, measured as the C-value, was observed in *T.stenocephalum* Boissier et Kotschy ex Boissier, 1875 (Trávníček et al. 2013) and in a sample of an unidentified species of the *Taraxacumofficinale* group in North America (Iaffaldano et al. 2017).

Taraxacum sect. *Taraxacum* (formerly known as T.sect.Ruderalia; generally known as *Taraxacumofficinale* group; see Kirschner and Štěpánek 2011) has a strongly prevailing triploid ploidy level of 2n = 3x = 24, by which it differs from other closely related sections (*Erythrosperma*, *Palustria*, and *Celtica*) with known ploidies of 3x and 4x or even higher. In this study, we aimed to count the chromosome number of 28 species for which knowledge was lacking and to detect the ploidy level for these species. Furthermore, we searched for variability in genome size among these species to determine whether we can detect variation in DNA content among species similar to that found in a sample of unidentified taxa of *T.officinale* group.

## Material and methods

### Plant Material

We studied a total of 28 *Taraxacum* species (25 formally described and three still undescribed, referred to by their working names) belonging to Taraxacum sect. *Taraxacum* (Table 1). Plants and achenes of the investigated species were collected in natural habitats of several localities of Central Europe in the period 2014–2016. A detailed description of the localities, date, and collectors of samples is provided in Table 1. The studied plant material was documented by herbarium specimens and is deposited in the herbarium of the Department of Botany, Palacký University in Olomouc, Czech Republic (OL). All studied species are apomictic (agamospermous); thus, maternal plants and offspring plants (grown from seeds) are taxonomically (genetically) identical.

**Table 1.** List of species used in this study, with sampling details. Country codes according to ISO 3166-1 alpha-2 (AT = Austria; CZ = Czechia, DE = Germany, HU = Hungary, IT = Italy, SK = Slovakia); Collectors: BT = Bohumil Trávníček; RJV = Radim Jan Vašut.

Taxon	Country	Locality; GPS; Date; Collector
*T.aberrans* Hagendijk, Soest & Zevenbergen, 1974	AT	Upper Austria, Obernberg am Inn town, lawn in the street of Therese-Riggle-Strasse; 48°19'14"N; 13°19'52"E; 10.05.2015; BT
*T.atroviride* Štěpánek & Trávníček, 2008	AT	Altaussee village (near Bad Aussee town), lawns and roadsides in the ski resort NNW from the village (valley of Augstbach brook); 47°39'42"N; 13°44'38"E; 08.05.2014; BT
*T.atrox* Kirschner & Štěpánek, 1997	IT	Cave del Predil settlement (S from Tarvisio town), lawns at the road no SP76 (at lake of Lago di Predil); 46°25'11"N; 13°33'42"E; 16.05.2015; BT
*T.baeckiiforme* Sahlin, 1971	HU	Felsöcsatár village (W from the Szombathely town), grassy roadsides at the road towards Vaskeresztes village; 250 m a.s.l.; 47°12'20"N; 16°26'51"E; 26.04.2015; BT
*T.chrysophaenum* Railonsala, 1957	CZ	Bartošovice village (near Nový Jičín town), lawns in park in central part of the village; 49°40'15"N, 18°02'59"E; 23.04.2014; BT
*T.coartatum* G. E. Haglund, 1942	CZ	Lubná village (near Polička town), grassy places at brook in E part of the village; 480 m a.s.l.; 49°46'26"N, 16°13'57"E; 17.05.2016; BT & RJV
*T.corynodes* G. E. Haglund, 1943	CZ	Hanušovice town, lawns at the railway station; 50°04'18"N, 16°55'52"E; 19.05.2015; BT
*T.crassum* H. Øllgaard & Trávníček, 2003	CZ	Nové Město na Moravě town, grassy places at brook in the town, ca 0.6 km ESE from railway station of “Nové Město na Moravě-zastávka”; 600 m a.s.l.; 49°33'45"N, 16°04'04"E; 17.05.2016; BT & RJV
*T.deltoidifrons* H. Øllgaard, 2003	CZ	Jimramov town, grassy places in the park of Bludník in N part of the town; 500 m a.s.l.; 49°38'19"N, 16°13'25"E; 17.05.2016; BT & RJV
*T.diastematicum* Marklund, 1940	CZ	Svratka village, meadows and grassy places at NW margin of the settlement of Česká Cikánka; 630 m a.s.l.; 49°42'35"N, 16°03'01"E; 17.05.2016; BT & RJV
*T.gesticulans* H. Øllgaard, 1978	CZ	Hanušovice town, lawns at the railway station; 50°04'18"N, 16°55'52"E; 19.05.2015; BT
*T.glossodon* Sonck & H. Øllgaard, 1999	CZ	Studnice village (N from Nové Město na Moravě town), meadow at road near the Paseky settlement ca 1 km NNW from the village; 780 m a.s.l.; 49°36'51"N, 16°05'17"E; 17.05.2016; BT & RJV
*T.guttigestans* H. Øllgaard in Kirschner & Štěpánek, 1992	CZ	Nové Město na Moravě town, grassy places at brook in the town, ca 0.6 km ESE from railway station of “Nové Město na Moravě-zastávka”; 600 m a.s.l.; 49°33'45"N, 16°04'04"E; 17.05.2016; BT & RJV
*T.huelphersianum* G. E. Haglund, 1935	CZ	Pekařov settlement (near Hanušovice town), lawns and meadows in the settlement; 50°04'41"N, 17°01'31"E; 19.05.2015; BT
*T.ingens* Palmgren, 1910	CZ	Svratka village, meadows and grassy places at NW margin of the settlement of Česká Cikánka; 630 m a.s.l.; 49°42'35"N, 16°03'01"E; 17.05.2016; BT & RJV
*T.jugiferum* H. Øllgaard, 2003	CZ	Jedlí village (NW from Zábřeh town), lawns and roadsides in central part of the village; 49°55'54"N, 16°47'45"E; 19.05.2015; BT
*T.laticordatum* Marklund, 1938	CZ	C Moravia, Hlinsko pod Hostýnem village, roadside at road towards Prusinovice village; 49°22'34"N; 17°36'47.8"E; 20.05.2016; BT
*T.lojoense* H. Lindberg, 1944 †	CZ	Úterý village (near Konstantinovy Lázně town), lawns at the brook on the eastern village margin; 510 m a.s.l.; 49°56'24"N, 13°00'21"E; 25.04.2014; BT
*T.lucidifrons* Trávníček, ineditus	CZ	Kunín village (near Nový Jičín town), lawns in chateau park; 49°38'39"N, 17°59'18"E; 23.04.2014; BT
*T.obtusifrons* Marklund, 1938	CZ	Lubná village (near Polička town), grassy places at brook in E part of the village; 480 m a.s.l.; 49°46'26"N, 16°13'57"E; 17.05.2016; BT & RJV
*T.ochrochlorum* G. E. Haglund, 1942	CZ	Svratka village, meadows and grassy places at NW margin of the settlement of Česká Cikánka; 630 m a.s.l.; 49°42'35"N, 16°03'01"E; 17.05.2016; BT & RJV
*T.ohlsenii* G. E. Haglund, 1936	DE	Schönwald village (near Hof town), wet meadow and adjacent roadsides at the road (no. 15) towards Rehau village; 550 m a.s.l.; 50°13'37"N, 12°04'57"E; 27.04.2014; BT
*T.perdubium* Trávníček, ineditus	CZ	Záhlinice village (near Hulín town), wet meadow 1.3 km SSW from the railway station; 190 m a.s.l.; 49°16'52"N, 17°28'58"E; 20.04.2016; BT
*T.praestabile* Railonsala, 1962	IT	Sella Nevea settlement (SW from Tarvisio town), lawns near hotel of Canin, road no. SP76; 46°23'19"N, 13°28'25"E; 16.05.2015; BT
*T.sepulcrilobum* Trávníček, ineditus	CZ	Záhlinice village (near Hulín town), wet meadow 1.3 km SSW from the railway station; 190 m a.s.l.; 49°16'52"N, 17°28'58"E; 20.04.2016; BT
*T.sertatum* Kirschner, H. Øllgaard & Štěpánek, 1997	CZ	Svratka village, meadows and grassy places at NW margin of the settlement of Česká Cikánka; 630 m a.s.l.; 49°42'35"N, 16°03'01"E; 17.05.2016; BT & RJV
*T.subhuelphersianum* M. P. Christiansen, 1971	SK	Spišské Podhradie village (near Levoča town), lawn at road not far from Sivá brada travertine spring; 49°00'28"N, 20°43'26"E; 01.05.2014; BT
*T.valens* Marklund, 1938	HU	Szombathely town, lawns in the Szent István park (at the street of Jókai Mór); 225 m a.s.l.; 47°13'45"N, 16°36'15"E; 26.04.2015; BT

† The taxon traditionally identified as *T.lippertianum* Sahlin, 1979 in Central Europe and recently considered a synonym of *T.debrayi* Hagendijk, Soest & Zevenbergen, 1972. According to BT, both taxa are synonyms of *T.lojoense* (B. Trávníček unpubl., H. Øllgaard pers. comm.).

For karyological analyses, achenes were sown in Petri dishes containing 1% agar solution and germinated at room temperature. Fresh young leaves for flow cytometric analyses were collected from juvenile plants cultivated in a greenhouse at the Department of Botany, Faculty of Science, Palacký University in Olomouc.

### Karyology

For chromosome counts, we used mitotically active root tip meristems of dandelion seedlings. Seedlings of the investigated species with 1–2 cm long roots were collected in the morning. To obtain the desired metaphase index, the roots were pre-treated in a 2 mM solution of 8-hydroxyquinoline for two hours at room temperature and an additional two hours at 4 °C in the dark. Then, the material was fixed in Carnoy’s fixative (a mixture (3:1, v/v) of absolute ethanol and acetic acid) and stored in a refrigerator (4 °C) until further processing (Hasterok and Maluszynska 2000). For slide preparation, a combination of protocols in Hasterok and Maluszynska (2000) and van Baarlen et al. (2000) was used with the following changes for the investigated species of dandelions. Fixed root tips were washed in citrate buffer (0.01 M, pH 4.8) for 5 min and then enzymatically digested in a mixture of 0.1% cellulose Onozuka RS (*Trichoderma* Persoon, 1794; Sigma), 0.1% pectolyase (*Aspergillusjaponicus* Saito, 1906; Sigma) and 0.1% cytohelicase (*Helixpomatia* Linnaeus, 1758; Sigma) in the citrate buffer for 90 min at 37–40 °C. To remove trace amounts of the enzymatic mixture, the root tips were then gently washed in citrate buffer for 5 min. Only the mitotically active meristematic tissue of a root tip was cut off under a stereoscopic microscope, transferred into a drop of 50% acetic acid on a slide and covered by a coverslip. After heating the preparation to 42 °C for 1–2 min, cells were spread between a glass slide and coverslip in a drop of 50% acetic acid. The coverslip was mechanically removed by a razor blade after deep freezing in liquid nitrogen, and the slide was air dried. To increase the contrast of metaphase chromosomes for counting, the preparations were stained with DAPI (4',6-Diamidine-2'-phenylindole dihydrochloride; Vectashield Mounting Medium with DAPI, Vector Laboratories). For each species, at least ten metaphases were analysed to determine the chromosome number. Well-spread metaphase images were captured using Olympus BX 60 and Axio Imager Z.2 Zeiss fluorescence microscopes, both equipped with a CCD camera and ISIS software (Metasystems, Altlussheim, Germany).

### Genome size estimation

The absolute genome size (2C-value; Doležel et al. 2007) of the fresh plant samples was quantified using a BD Accuri C6 flow cytometer (BD Biosciences, San Jose) equipped with a blue laser (488 nm, 20 mW, BD Accuri; BD Biosciences, San Jose). Sample preparation followed the standard protocol using LB01 isolation buffer supplemented with PVP (polyvinylpyrrolidone, 10 g/500 ml of buffer) to suppress interference of phenolic compounds with DNA staining (Doležel and Bartoš 2005, Doležel et al. 2007). Approximately 0.2 cm^2^ of the plant tissue between secondary veins was chopped in 500 μl of LB01 buffer together with a similar amount of tissue of an internal standard. Due to peak overlap in some accessions, *Solanumlycopersicum* Linnaeus, 1753 ‘Stupické polní rané’ (2C = 1.96 pg; Doležel et al. 2007) served as the primary reference standard, and *Glycinemax* (Linnaeus, 1753) Merrill, 1917 ‘Polanka’ (2C = 2.33 pg, re-calculated against a primary standard) served as the secondary standard. The suspension was filtered through a 42 μm nylon mesh, supplemented with 20 μl of RNase A type II-A (with a final concentration of 50 μg/ml) and incubated at room temperature for approximately 10 min. The sample was then stained with 20 μl of propidium iodide (PI; final concentration of 50 μg/ml) and incubated with occasional shaking for approximately 5 min at room temperature. A flow-through fraction was then run on the flow cytometer, and the relative fluorescence intensity of at least 5,000 particles was recorded. Each sample was analysed at least three times. If the range of variation in the three measurements exceeded the 2% threshold, then the outlying value was discarded, and the sample was re-analysed. Only G0/G1 peaks with coefficients of variation < 4% were accepted. The 2C-value was calculated by multiplying the 2C-value of the standard with the sample/standard fluorescence ratio. Monoploid genome size (1Cx-value) was calculated by dividing the 2C-value by the inferred chromosome number.

## Results

**Figure 1.**
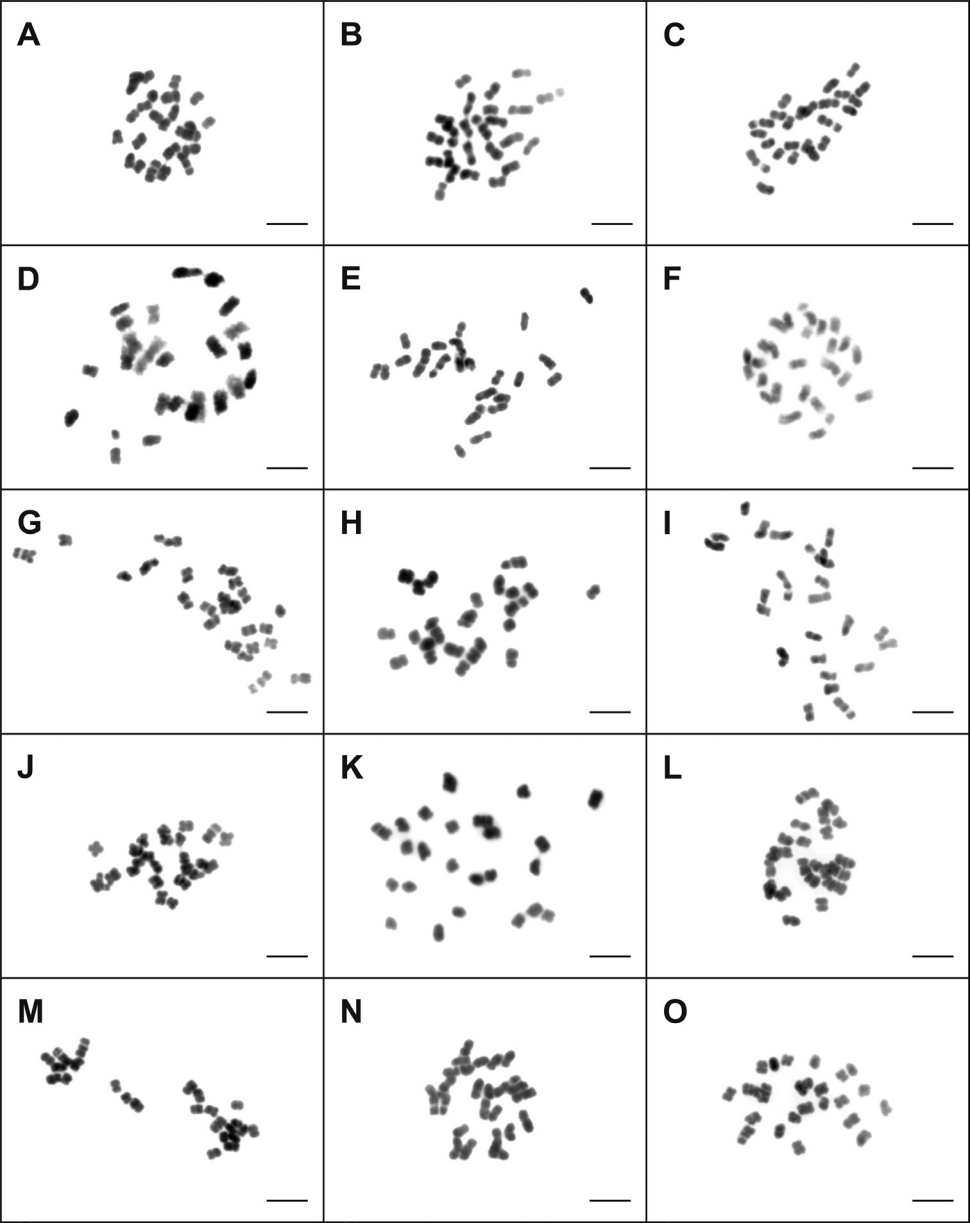
Mitotic metaphase chromosomes of studied triploid species (2n=3x=24) of Taraxacum sect. *Taraxacum*. **A***T.aberrans***B***T.atroviride***C***T.atrox***D***T.baeckiiforme***E***T.chrysophaenum***F***T.coartatum***G***T.corynodes***H***T.crassum***I***T.deltoidifrons***J***T.diastematicum***K***T.gesticulans***L***T.glossodon***M***T.guttigestans***N***T.huelphersianum***O***T.ingens*. Scale Bar: 5µm.

**Figure 2.**
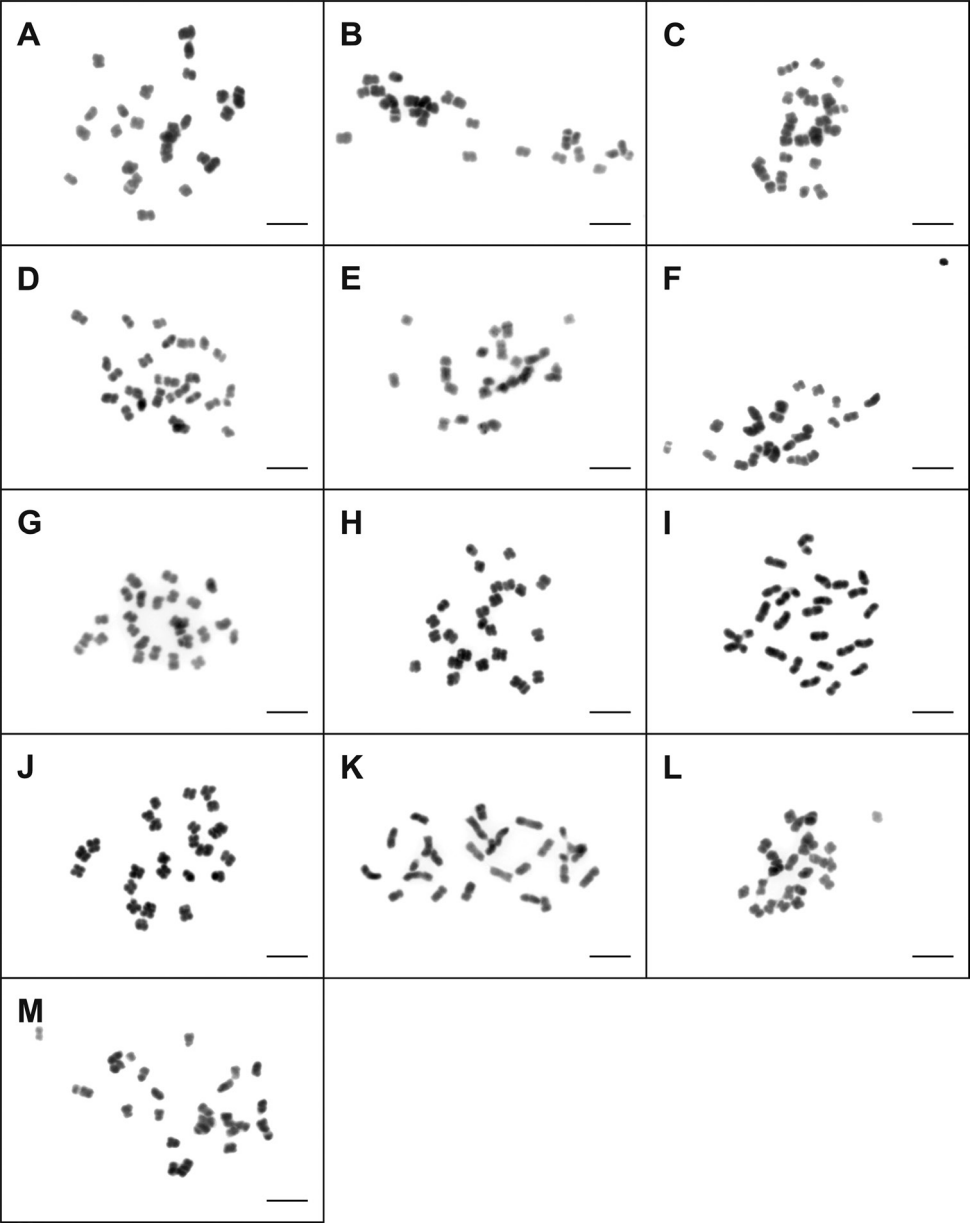
Mitotic metaphase chromosomes of studied triploid species (2n=3x=24) of Taraxacum sect. *Taraxacum*. **A***T.jugiferum***B***T.laticordatum***C***T.lojoense***D***T.lucidifrons***E***T.obtusifrons***F***T.ochrochlorum***G***T.ohlsenii***H***T.perdubium***I***T.praestabile***J***T.sepulcrilobum***K***T.sertatum***L***T.subhuelphersianum***M***T.valens*. Scale Bar: 5µm.

The chromosome number of all 28 studied species of Taraxacum sect. *Taraxacum* (*T.aberrans*, *T.atroviride*, *T.atrox*, *T.baeckiiforme*, *T.chrysophaenum*, *T.coartatum*, *T.corynodes*, *T.crassum*, *T.deltoidifrons*, *T.diastematicum*, *T.gesticulans*, *T.glossodon*, *T.guttigestans*, *T.huelphersianum*, *T.ingens*, *T.jugiferum*, *T.laticordatum*, *T.lojoense*, *T.lucidifrons*, *T.obtusifrons*, *T.ochrochlorum*, *T.ohlsenii*, *T.perdubium*, *T.praestabile*, *T.sepulcrilobum*, *T.sertatum*, *T.subhuelphersianum*, *T.valens*) was counted invariably as 2n = 3x = 24 (Figs 1, 2). With respect to the position of the centromere, the chromosomes of all studied species were predominantly sub-metacentric or metacentric. The chromosome sizes were relatively small (Figs 1, 2). The smallest chromosome size in this study was 1.02 µm (*T.ochrochlorum*), and the largest one was 4.94 µm (*T.baeckiiforme*).

The DNA content of the twenty-six studied *Taraxacum* species (two species, i.e., *T.chrysophaenum* and *T.subhuelphersianum*, were not analysed due to low-quality fresh material) ranged 1.08-fold from 2C = 2.60 pg in *T.atrox* to 2C = 2.86 pg in *T.perdubium* (Table 2). The average and median 2C-values for Taraxacum sect. *Taraxacum* (based on these 26 species) are 2.72 pg and 2.71 pg, respectively.

**Table 2.** Nuclear DNA content of studied Taraxacum sect. *Taraxacum* species (Lyc = *Solanumlycopersicon* ‘Stupické polní rané’; Gly = *Glycinemax* ‘Polanka’; n.a. = not analysed, N = number of plants analysed; 1Cx = monoploid genome size, 2C = DNA amount/ploidy level).

Species	2C DNA amount [pg] (mean ± s.d.)	N	Ploidy	1Cx [pg]	Standard
* T. aberrans *	2.71 ± 0.010	3	3x	0.90	Lyc
* T. atroviride *	2.70 ± 0.020	2	3x	0.90	Lyc
* T. atrox *	2.60 ± 0.002	2	3x	0.87	Lyc
* T. baeckiiforme *	2.62 ± 0	1	3x	0.87	Lyc
* T. chrysophaenum *	n.a.	n.a.	3x	n.a.	n.a.
* T. coartatum *	2.72 ± 0.070	2	3x	0.91	Lyc
* T. corynodes *	2.67 ± 0.001	2	3x	0.89	Lyc
* T. crassum *	2.62 ± 0.020	2	3x	0.87	Lyc
* T. deltoidifrons *	2.69 ± 0.007	3	3x	0.90	Lyc
* T. diastematicum *	2.67 ± 0	1	3x	0.89	Lyc
* T. gesticulans *	2.83 ± 0.040	2	3x	0.94	Lyc
* T. glossodon *	2.77 ± 0.010	2	3x	0.92	Lyc
* T. guttigestans *	2.74 ± 0.004	2	3x	0.91	Lyc
* T. huelphersianum *	2.79 ± 0.006	2	3x	0.93	Lyc
* T. ingens *	2.68 ± 0.013	3	3x	0.89	Gly + Lyc
* T. jugiferum *	2.71 ± 0.001	2	3x	0.90	Lyc
* T. laticordatum *	2.84 ± 0.008	2	3x	0.95	Lyc
* T. lojoense *	2.62 ± 0.020	4	3x	0.87	Lyc
* T. lucidifrons *	2.81 ± 0	1	3x	0.94	Lyc
* T. obtusifrons *	2.75 ± 0.03	2	3x	0.92	Lyc
* T. ochrochlorum *	2.67 ± 0	1	3x	0.95	Gly
* T. ohlsenii *	2.63 ± 0	1	3x	0.88	Lyc
* T. perdubium *	2.86 ± 0	1	3x	0.95	Lyc
* T. praestabile *	2.73 ± 0.050	3	3x	0.91	Lyc
* T. sepulcrilobum *	2.72 ± 0	1	3x	0.91	Lyc
* T. sertatum *	2.69 ± 0.010	2	3x	0.90	Lyc
* T. subhuelphersianum *	n.a.	n.a.	3x	n.a.	n.a.
* T. valens *	2.70 ± 0	1	3x	0.90	Lyc

## Discussion

Chromosome number variation differs among sections of the genus *Taraxacum* and more frequently occurs in sections such as *Palustria* or *Celtica*, whereas in section Taraxacum (and also section Hamata), it is nearly unknown. In our study, we aimed to either find variation in ploidy or confirm the prevailing triploid level. Our findings confirmed previously published records of 2n = 3x = 24 for *T.diastematicum* and *T.obtusifrons* (Uhlemann 2001, Salih et al. 2017); the chromosome numbers for all other 26 species are new findings. The ploidy level measured by flow cytometry was previously documented for 11 species (*T.atrox*, *T.baeckiiforme*, *T.corynodes*, *T.crassum*, *T.glossodon*, *T.guttigestans*, *T.ingens*, *T.laticordatum*, *T.ohlsenii*, *T.sertatum* and *T.valens*; Trávníček et al. 2010); we now provide exact information on chromosome numbers and genome size estimations.

A tetraploid chromosome number (2n = 4x = 32) was counted for only a few species of the 165 species of T. sect. *Taraxacum* with known chromosome numbers in the Chromosome Counts Database (CDDB, version 1.45; Rice et al. 2015). None of the records can be considered fully reliable due to frequent misidentifications of the *Taraxacum* microspecies (lack of identification by specialists). Den Nijs and Sterk (1984) published two chromosome counts, i.e., triploid (2n = 3x = 24) and tetraploid (2n = 4x = 32), for species named as *T.lacistrum* Sahlin, 1982, and collected in France; however, the tetraploid number is listed as a question mark, and this chromosome number must therefore be considered dubious (due to the apomictic behaviour of microspecies, it is implausible to have 2 different ploidy levels for the same species). The chromosome number for a species from the High Atlas, *T.atlantis-majoris* H. Lindberg, 1932 was counted as tetraploid, but the species identification is mentioned as “T.cf.atlantis-majoris”, and misidentification as other species (even from other sections, such as *Piesis*) cannot be excluded (Oberprieler and Vogt 1993). The tetraploid record for *T.albertshoferi* Sahlin, 1984 (Sahlin 1984) cannot be accepted without doubt either, because in the same paper, *T.franconicum* Sahlin, 1984 (which is now considered a synonym of *T.plumbeum* Dahlstedt, 1911) is also described with a tetraploid chromosome number, which was confirmed to be erroneous; the correct one is triploid (e.g., Vašut 2003). The tetraploid record for *T.mediterraneum* Soest, 1954 (Cardona and Contandriopoulos 1983; identified as *T.balearicum* Soest, 1961) does not refer how the taxon was determined. Rousi et al. (1985) published a tetraploid record for *T.penicilliforme* H. Lindberg, 1907 as a member of T.sect.Vulgaria (= T. sect. *Taraxacum*), but this species belongs to T.sect.Borea. Thus, the only somewhat reliable record of a tetraploid in Taraxacum sect. *Taraxacum* is for the alpine species *T.venticola* A. J. Richards, 1972 (Richards 1972).

Our list of species of T. sect. *Taraxacum* mainly includes typical members of the section, which differ slightly in their eco-geographic preferences. Some species have (in Central Europe) a preference for wet and sub-oceanic regions (such as *T.corynodes*, *T.chrysophaenum*, *T.lucidifrons* and *T.ochrochlorum*); on the other hand, some occupy more xerothermic regions (e.g., *T.atrox*, *T.baeckiiforme*, and *T.lojoense*). Some species resemble members of T.sect.Celtica (*T.lucidifrons*) or T.sect.Palustria (*T.perdubium* and *T.sepulcrilobum*). However, although the species in our study differ somewhat in ecology and geography, there is no variation in their ploidy levels. This is in agreement with previous studies in which only a triploid level was undoubtedly recorded for Nordic (“Atlantic”) and Pannonian or Mediterranean species.

Genome size estimates in Taraxacum sect. *Taraxacum* are very limited. Only a few papers dealt with its genome size (Bennett et al. 1982, Záveský et al. 2005, Bainard et al. 2011, Iaffaldano et al. 2017), but none of these papers studied known apomictic microspecies; only unknown species of the *T.officinale* group were measured. Generally, the genome size of the *T.officinale* group varies between 2C = 1.65 pg and 2C = 3.09 pg (Bennett et al. 1982, Záveský et al. 2005, Vidic et al. 2009, Temsch et al. 2010, Bainard et al. 2011, Iaffaldano et al. 2017; summarized in Table 3); values between 2C = 1.65–1.74 pg (Záveský et al. 2005, Iaffaldano et al. 2017) are equal to a diploid ploidy level (i.e., the species *T.linearisquameum*). The genome size of triploid apomicts apparently ranges from 2C = 2.45 pg to 2.76 (3.09) pg (see literature above). Our results are among the highest recorded values. The overall variation in recorded values is approximately 16 % (excluding the highest value of 2C = 3.09 pg, which may represent an aneuploid or tetraploid plant). Such variation can reflect real genome size variation among different species (individuals). Within a single species, *Taraxacumstenocephalum* (T.sect.Piesis), an ~1.2-fold difference in DNA content is documented (1.194-fold difference for DAPI and 1.219-fold difference for PI; Trávníček et al. 2013). Greater variation in DNA content can be attributed to the sexual reproduction of the species (in contrast to the apomictic reproduction of the species in our study). Even greater variation in DNA content was documented in *Picrishieracioides* Linnaeus, 1753 (Asteraceae, Cichorioideae, Cichorieae); in diploid sexual species, it ranged from 2C = 2.26 to 3.11 pg (1.37-fold difference; Slovák et al. 2009). In other genera of Asteraceae with the occurrence of apomictic taxa, such as *Hieracium* Linnaeus, 1753 and *Pilosella* Hill, 1756 DNA content variation is considerably larger than the known variation in Taraxacum sect. *Taraxacum*, i.e., 2.37-fold and 4.3-fold, respectively (Suda et al. 2007, Chrtek et al. 2009).

**Table 3.** Genome size estimates of *T.officinale* group in literature record. Values with asterisk (*) indicate re-calculated values according to conversion rate of 1 pg ~ 9.78×10^8^ bp (Doležel et al. 2003).

**Literature**	**2C [pg**]	**2C [Gbp**]
Bennett et al. 1982	2.55	2.49*
Záveský et al. 2005	1.74–2.70	1.70–2.64*
Vidic et al. 2009	2.56*	2.50
Temsch et al. 2010	2.51	2.45*
Bainard et al. 2011	2.67	2.61*
Iaffaldano et al. 2017	1.65–3.09* (2.45–2.76*)	1.61–3.02 (2.40–2.70)
this study	2.60–2.86	2.54–2.80*

Genome size estimates vary in all taxa. Multiple factors can affect the measurement of genome size, e.g., differences in instrument settings among the instruments used (Doležel et al. 1998), using inadequate dye (DAPI vs. PI; Doležel et al. 1992), interactions between the dye and other molecules that lead to cytosolic effects (Noirot et al. 2000), and discrepancies in standardization (Doležel and Greilhuber 2010). Applying different laboratory procedures to the same species can lead to up to <10% variation; in the *T.officinale* group, different treatments led to a difference of up to 8.7% (Bainard et al. 2011). Therefore, at least part of the difference among published records can be attributed to a bias due to differences in laboratory procedures. We used a standardized procedure (buffers, tissue treatments, etc.) in our lab; therefore, the observed variation among the species used in this study likely reflects the real variation in DNA content.

Our study provided new data for 26 species of T. sect. *Taraxacum*, which confirmed no variation in chromosome number and ploidy level (2n = 3x = 24) and revealed only minor variation in DNA content that roughly equalled a possible methodological bias. The species sampled cover variation within the section: a sample of typical T. sect. *Taraxacum* species (most of the studied species) but also species that by morphology or ecology are intermediates of other sections, i.e., *T.perdubium* and *T.sepulcrilobum*, which are morphological and ecological intermediates between the studied section and T.sect.Palustria; or *T.lucidifrons*, which is morphologically similar to T.sect.Celtica or species resembling members of T.sect.Borea (*T.ohlsenii*, *T.lojoense* and *T.atrox*). Two species in our list are apolliniferous (*T.atrox* and *T.subhuelphersianum*). Such unusual homogeneity among species in T. sect. *Taraxacum* rather than great morphological (and ecological) variability might reflect a young evolutionary origin, which is likely in contrast to sections *Palustria*, *Erythrosperma* and others that may partly consist of evolutionarily older species (Wittzell 1999, Majeský et al. 2012, Kirschner et al. 2015). Although there is no evidence for the potential evolutionary scenario in European *Taraxacum* sections, we can speculate that the origin of apomictic species of T. sect. *Taraxacum* (*T.officinale* group) may be a result of “recent” hybridization between triploid apomicts and diploid sexuals in the sexual-asexual cycle in a mixed dandelion population, a phenomenon experimentally described in this group (Tas and van Dijk 1999, van Dijk 2003, van Dijk and Vijverberg 2005). In a mixed population (2x and 3x cytotypes; sexual and apomictic types), triploids are results of hybridization between triploid apomicts (diploid pollen) and diploid sexuals (haploid egg cell); however, a rare occurrence of tetraploidy (probably of temporary occurrence) can accelerate the formation of novel triploids (Verduijn et al. 2004b). These tetraploids probably occur in nature as a (rare) product of hybridization in mixed populations (probably discovered in the papers of Sato et al. 2014 or Iaffaldano et al. 2017; Ľ. Majeský, unpublished results) and function as a bridge in the formation of novel stable apomictic microspecies, but probably no such temporary tetraploid hybrids evolved in stable microspecies.
